# Exploring Medical Students' Knowledge and Perceptions of Physical Medicine and Rehabilitation Specialty in Qassim University

**DOI:** 10.7759/cureus.67300

**Published:** 2024-08-20

**Authors:** Ahmad H Al-Washmi, Noura I Al-Otayk, Nawaf S Al-Rubaysh, Samar A Al-Harbi, Faisal T Al-Ayed, Rayan S Al-Khedhairi, Abdulmajeed E Al-Harbi, Khalid M Al-Khalifah

**Affiliations:** 1 Department of Orthopedic Surgery, College of Medicine, Qassim University, Buraydah, SAU; 2 College of Medicine, Qassim University, Buraydah, SAU; 3 Department of Physical Medicine and Rehabilitation, King Fahad Specialist Hospital, Buraydah, SAU; 4 College of Medicine, Qassim University, Unaizah, SAU

**Keywords:** higher education, perception, health knowledge, students, cross-sectional, physical medicine and rehabilitation

## Abstract

Objectives*:* Physical Medicine and Rehabilitation (PM&R) is a medical branch that aims to manage, prevent, and diagnose people who are disabled due to disease, disorder, or injury. The purpose of this study is to explore the level of awareness and scope of the PM&R specialty among medical students.

Methods: A cross-sectional study was carried out at Qassim University, Saudi Arabia, with 287 medical students. Cochran's method was used to establish the sample size of 287 medical students with a 5% margin of error and a 95% confidence level. An online survey was conducted among undergraduate students at Qassim University's Medical College. SPSS was utilized to study the characteristics of the population demographics. An independent t-test was used to calculate the significant values of each questionnaire. A significant value of <0.05 was considered as a significant value.

Results*:* Among 287 medical students, 38.5% of students agreed to PM&R course inclusion in the undergraduate curriculum. 78.8% of students agreed with no specialization in PM&R, and 60.8% agreed that the lack of PM&R in Saudi area hospitals contributed to less recognition. Overall, medical students had low to moderate knowledge of PM&R.

Conclusion*:* The study presents the need to train medical students in PM&R during their medical studies, develop more recognition, and integrate musculoskeletal and physical checkup skills among medical students. Moreover, it should have a practical implication across the nation so that more medical students develop knowledge and skills.

## Introduction

Physical Medicine and Rehabilitation (PM&R), often referred to as physiatry, is a medical specialty that focuses on diagnosing, evaluating, and treating a wide range of medical conditions that affect the musculoskeletal system, nervous system, and overall physical functionality [1-2]. The primary goal of PM&R is to enhance and restore a patient's functional ability and quality of life, emphasizing non-surgical methods whenever possible. The increasing occurrence of accidents, cardiovascular events, and cancer cases, the primary contributors to disabilities, underscores the critical need to focus on rehabilitation programs [3]. Positioned as a pivotal component of comprehensive patient care, rehabilitation medicine places significant emphasis on providing ongoing support to patients, taking into account not only their physical needs but also their psychological and social challenges [4].

Unlike other medical specialties, the emphasis on PM&R is not uniformly integrated into all medical school curricula. The rising population of individuals with disabilities has often faced suboptimal care and support tailored to their unique needs. This inadequacy can be attributed to a deficiency in understanding the comprehensive scope of practice within PM&R and a dearth of exposure to the multidisciplinary approach required for holistic patient care. To illustrate, fewer than 50% of medical schools in the United States possess a dedicated academic PM&R Department, and the proportion with PM&R residency programs is even smaller [5].

The Association of Academic Physiatrists put forth recommendations concerning undergraduates' education in managing patients with chronic conditions and disabilities in 2007 [6]. As outlined in the 2010 report from the British Society of Rehabilitation Medicine, it is recommended that rehabilitation education be delivered through a structured course facilitated by specialists in PM&R [7]. Starting from 1987 in the UK and spanning to 2017 in Germany, a range of distinct programs in PM&R were developed for medical students [8-9]. These programs encompassed various educational elements, including lectures, seminars, hands-on physical examination sessions, multidisciplinary rehabilitation clinic experiences, and specialized modules covering orthotics, prosthetics, and various physical modalities. Anticipated outcomes of these programs included increased satisfaction, enhanced accuracy in rehabilitation knowledge, and improved attitudes among participating students. Consequently, a portion of these students opted for PM&R as their specialty of choice [10]. Experts from the Christiana Care Health System proposed that it is equally important to educate primary care physicians about the advantages of referring patients to physiatrists [11].

There is a paucity of research focused on evaluating the extent of medical students' knowledge concerning PM&R. Yorke et al. highlighted the importance of assessing and enhancing medical students' attitudes toward physical medicine to enhance patient care within this domain [12]. Faulk et al. conducted a two-year study that assessed the impact of a two-week mandatory rotation in PM&R on fourth-year medical students' attitudes towards teamwork and knowledge of PM&R involving 138 medical students implemented a two-week curriculum, assessing pre- and post-test attitudes and knowledge towards PM&R. Around 76% of participants recognized the significance of musculoskeletal education, with 42% suggesting a longer duration for the clinical curriculum [13]. Emami Razavi et al. evaluated an assessment of an innovative undergraduate PM&R course at Tehran University of Medical Sciences and revealed significant improvements in students' attitudes. Participants acknowledged the course's value and preferred referring musculoskeletal patients to physiatrists. Attitude positively correlated with practice scores, indicating a predictive solid relationship. The study suggests that such courses can effectively enhance PM&R education, impacting career choices and patient referrals [14].

In a context where the availability of PM&R programs is limited within the country, assessing whether students recognize the value of such rotations for their educational journey and whether they contribute to enhancing their knowledge and attitudes becomes crucial. The primary aim of this study is to explore the level of awareness and scope of the PM&R specialty among medical students at Qassim University.

## Materials and methods

Study design and population

The study design of this research is cross-sectional. The targeted population was the medical students of College of Medicine at Qassim University in Qassim, Saudi Arabia.

Targeted area

The study was conducted at the College of Medicine at Qassim University in Qassim, Saudi Arabia.

Sampling and sample size

The sample was obtained from 393 medical students at Unaizah Medical College and 726 medical students at Qassim College of Medicine “main campus”. Cochran's method was used to establish the sample size of 287 medical students from Buraidah and Unaizah Medical Colleges, with a 5% margin of error and a 95% confidence level.

Inclusion and exclusion criteria

The inclusion criteria were undergraduate students at the Medical College of Qassim University, and the exclusion criteria were postgraduate students and students who did not respond to all survey elements or demographic data.

Data sources and collection

The questionnaire was designed following an intensive literature review and consultation with experts in PM&R and medical education. The questionnaire items were adapted from previously validated instruments used in similar studies to ensure relevance and comprehensiveness. Data was collected via an online questionnaire link distributed to undergraduate medical students at Qassim University's Medical College.

Reliability and validity assessment

While specific reliability and validity tests were not conducted for this study, the questionnaire items were based on validated instruments from previous research. Expert consultation was utilized to ensure the content validity of the questionnaire. Future research could benefit from conducting a pilot study and psychometric evaluation to further establish the reliability and validity of the instrument.

Justification for not conducting a pilot study

A pilot study was not conducted due to time constraints and logistical challenges. Instead, expert feedback was sought to refine the questionnaire, ensuring clarity, relevance, and comprehensiveness of the items. This approach was deemed sufficient to achieve the study objectives within the given constraints.

Statistical analysis

All the collected data from the questionnaire was coded and verified before data entry. IBM SPSS Statistics for Windows, Version 25 (Released 2017; IBM Corp., Armonk, New York, United States) allowed data entry and statistical analysis. Statistical significance is determined using the significance difference, either equal to or smaller than 0.05. Using SPSS, descriptive statistics are presented, including frequency, mean, median, standard deviation, P-value, and the percentage of respondents. In addition, tests of significance, such as the Chi-test and and independent sample t-test, are applied. The 0.05 value of the results is considered a statistically significant value. Moreover, a 95% confidence interval is also determined.

Data management

Data management involved secure storage of completed questionnaires, with immediate coding for anonymity. A double-entry verification process minimized errors and prevented data loss. Missing data is addressed ethically, either through imputation or exclusion. Access is restricted to authorized personnel, with confidentiality agreements and security measures in place to protect data integrity. An audit trail tracked modifications for transparency. At the end of the study, the dataset is archived securely for future use. This systematic approach ensures data accuracy, confidentiality, and accessibility throughout the research process.

Ethical considerations

All participants are informed that their participation is voluntary, and their consent is obtained before their participation. Before distributing the questionnaire, the purpose of the research and its significance in making healthcare policies are explained to the participants to avoid complications. In addition, their confidential data is secured and kept private. Ethical approval of this research is approved by the Committee of Research Ethics, Deanship of Scientific Research, Qassim University, with IRB approval number [H-04-Q-001], on 26 October 2023.

Study duration

The study was carried out from October 2023 to November 2023.

## Results

Table 1 provides a detailed analysis of medical students' opinions on many facets of PM&R based on a thorough survey with 287 respondents. The information gathered from a wide range of samples provides insightful information about how well students comprehend PM&R and its potential as a medical specialty. There is a balanced representation in the demographic distribution, with 52.8% women and 46.9% men among the responders. A diversified student body is reflected in the distribution across academic years and medical intern status, with the majority (58.3%) falling within the 22-25-year age range (see Table 1).

**Table 1:** Demographic characteristics of respondents

Variables	Frequency (n= 287)	Percentage (%)
Gender		
Male	135	46.9
Female	152	52.8
Age		
18-21	92	31.9
22-25	168	58.3
26-30	27	9.4
Year of Study		
1	48	16.7
2	38	13.2
3	42	14.6
4	79	27.2
5	57	19.8
Medical intern	23	8.0

A significant percentage of participants (60.1%) expressed familiarity with the PM&R specialty, suggesting a notable level of awareness within the sample. However, a sizable portion (39.6%) continued to be ignorant, indicating a possible need for more education and awareness campaigns. The majority of students showed a diverse but usually moderate level of awareness when it came to PM&R sub-specialties, frequently addressed conditions, and PM&R's involvement in patient care. It's interesting to note that a sizable percentage of respondents (84.4%) said they had no official education or training in PM&R during their medical studies, which raises the possibility of a curriculum gap. Students' ability to distinguish between physical therapy and PM&R varied, and their favored methods of approaching a PM&R specialist revealed a range of perspectives. There was a reasonable distribution in the knowledge of the clinical contexts in which PM&R practices were used, with a significant percentage (39.9%) recognizing its application in a variety of healthcare contexts (see Table 2).

**Table 2:** Evaluation of medical students' perspectives on Physical Medicine and Rehabilitation (PM&R)

Variables	Frequency (n= 287)	Percentage (%)
Are you familiar with the specialty of Physical Medicine and Rehabilitation (PM&R)?		
Yes	173	60. 1
No	114	39.6
PM&R sub-specialities, etc.:		
High	38	13.2
Low	81	28.2
Moderate	61	21.3
Very high	27	9.4
Very low	80	27.8
Common conditions treated in PM&R:		
High	58	20.1
Low	59	20.5
Moderate	87	30.2
Very high	21	7.3
Very low	62	21.5
PM&R's role in patient care and recovery:		
High	86	29.9
Low	55	19.1
Moderate	65	22.6
Very high	48	16.7
Very low	33	11.5
Have you received formal education or training in PM&R during your medical studies?		
Yes	44	15.3
No	243	84.4
Do you differentiate between PM&R and Physical Therapy?		
Yes	86	29.9
No	201	69.8
How would you address a specialist in PM&R?		
Not sure	89	30.9
Orthopedician	32	11.1
Physiatrist	26	9.0
Physiotherapist	87	30.2
Rehabilitist	53	18.4
In which clinical setting do you believe PM&R practices?		
All of the above	115	39.9
Consultations	35	12.2
Inpatient settings	11	3.8
Interventional sessions	38	13.2
Not sure	41	14.2
Outpatient clinics	47	16.3
Which demographics do you believe require the services of PM&R?		
Adults	24	8.3
Children	2	7
Elderly	27	9.4
Outpatient clinics	33	11.5
All of the above	201	69.8
Which conditions do you think PM&R physicians address?		
Amputations	1	0.3
Pain management	26	9.0
Spinal cord injury	15	5.2
Sports medicine	33	11.5
Stroke	24	8.3
All of the above	188	65.3
Do you understand the patient population that PM&R serves?		
Yes	124	43. 1
No	57	19.8
Not sure	72	25.0
All of the above	34	11.8
In which bodily system is PM&R most concerned?		
Cardiovascular system	1	0.3
Central nervous system	47	16.3
Gastrointestinal tract	1	0.3
Musculoskeletal	103	35.8
Not sure	63	21.9
All of the above	72	25.0
Do you believe PM&R can enhance patients' quality of life?		
Yes	238	17.0
No	49	82.6
Do you agree that every multispeciality hospital should have a PM&R Physician?		
Yes	242	84.0
No	45	15.6
Do you intend to specialize in Physical Medicine and Rehabilitation in the future?		
Yes	61	21.2
No	227	78.8

Additionally, respondents' views on the demographics in need of PM&R services varied, with the majority (69.8%) supporting the necessity of PM&R services for all age groups and demographics. 64.6% of respondents agreed that the availability of PM&R services without a referral is an essential component of patient care. The fact that students could distinguish between a physiatrist, podiatrist, and psychiatrist, as well as the fact that PM&R treats a broad range of ailments, such as pain management, spinal cord injuries, sports medicine, stroke, and amputations, are noteworthy findings. A significant number of students acknowledged uncertainty regarding their comprehension of the patient population that PM&R serves, with various degrees of confidence voiced by the students. In a similar vein, differing viewpoints within the student body were indicated by the opinions expressed regarding the body system that PM&R primarily concerns itself with. The views of the students regarding the inclusion of PM&R material in the undergraduate curriculum are also illuminated by the table. There was a range of opinions among the respondents: 38.5% supported its inclusion, 23.6% opposed it, and 37.5% were unsure (see Table 3).

**Table 3:** Perceptions and awareness of Physical Medicine and Rehabilitation (PM&R): survey responses among medical students

Description	Yes n (%)	No n (%)	Not sure n (%)
Can patients of all ages consult a PM&R specialist?	186 (64.6)	31 (10.8)	70 (24.3)
Do you think a referral from another doctor is necessary to see a PM&R physician?	112 (38.9)	66 (22.9)	109 (37.8)
Is there a distinction between a Physiatrist, Podiatrist, and Psychiatrist?	140 (48.6)	30 (10.4)	117 (40.6)
Do you believe PM&R only deals with patients who have already been diagnosed?	128 (44.4)	67 (23.3)	92 (31.9)
Should the undergraduate curriculum include content on PM&R?	111 (38.5)	68 (23.6)	108 (37.5)
Is there potential for PM&R to be a specialty in your country?	149 (51.7)	31 (10.8)	107 (37.2)
Do you consider PM&R a well-known speciality among the medical population?	93 (32.3)	114 (39.6)	80 (27.8)
Is there a strong connection between undergraduate exposure and postgraduate plans involving PM&R?	144 (50.0)	45 (15.6)	98 (34.0)
Could the lack of PM&R departments and physicians in your area's hospitals contribute to its lesser recognition?	175 (60.8)	26 (9.0)	86 (29.0)

**Figure 1:**
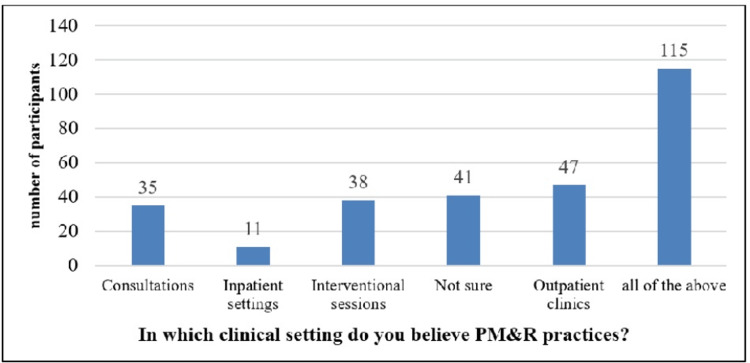
Graphical presentation of the participant’s response to the question; in which clinical setting do you believe PM&R practices?

The responses of participants to the question "In which clinical setting do you believe PM&R practices?" were represented in the bar chart (Figure 1). The six groups on the graph corresponded to distinct clinical settings: consultations, outpatient clinics, inpatient settings, interventional sessions, not sure, and all of the above. 35, 11, 38, 47, 41, and 115 individuals, respectively, believed that PM&R (Physical Medicine and Rehabilitation) practices existed in each context. It was observed that "all of the above" was the category with the greatest number of participants (115), suggesting that the majority of participants thought PM&R procedures took place in all of the clinical settings listed. This is followed by 47 participants in outpatient clinics, 41 participants in not sure, 38 participants in interventional sessions, 35 participants in consultations, and 11 individuals in inpatient settings. This information offers a fascinating look into how people perceive the locations of PM&R activities (Figure 1).

**Figure 2:**
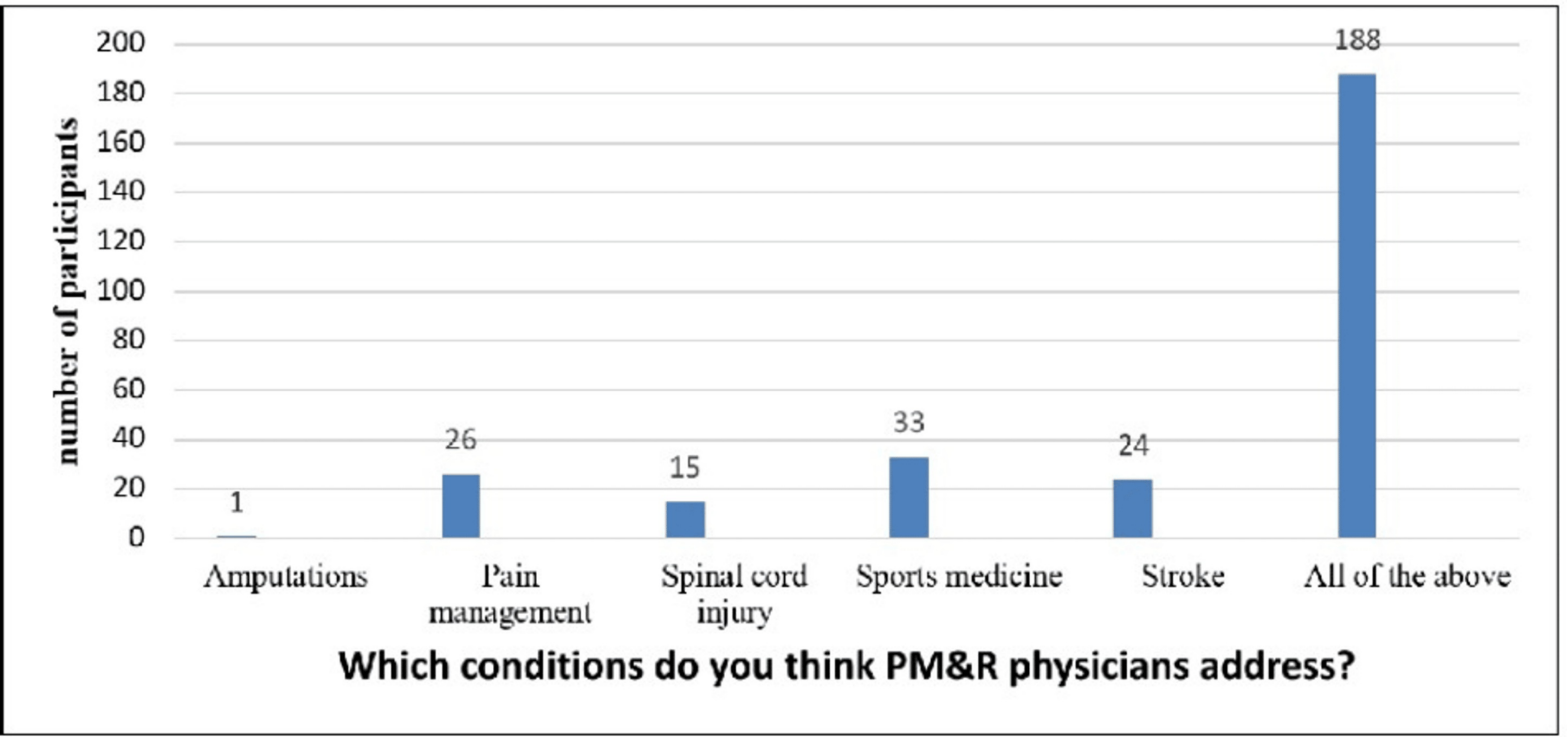
Graphical presentation of the participant’s response to the question ‘which conditions do you think PM&R physicians address’?

This graph was a representation of the participants' answers to this query "Which conditions do you think PM&R physicians address?”. Six distinct groups on the chart each denoted a distinct medical problem. Amputations, pain management, stroke, spinal cord injuries, sports medicine, and all of the above were the categories under question. Participants were divided into 1, 26, 15, 33, 24, and 188 groups who thought that PM&R doctors handled each ailment. "All of the above" was the group with the most participants (188), suggesting that most participants thought PM&R doctors treated every illness on the list. Sports medicine had 33 individuals, pain management had 26, stroke had 24 people, spinal cord injury had 15 participants, and amputations had one person afterward. This information offered an intriguing look into how people perceive the problems that PM&R doctors treat (Figure 2).

Table 4 provided crucial context for understanding knowledge about PM&R. The median value of 4.00 suggested that respondents generally held positive opinions about PM&R sub-specialties, common conditions treated in PM&R, and the function of PM&R in patient care and recovery. Positive perceptions were substantiated by the mean values with standard deviations, which demonstrated a statistically significant difference for each category (p-values < 0.05), indicating a consensus among the respondents. Furthermore, with a mean value of 2.15 ± 0.367 and a significant p-value of 0.000, the data indicated that respondents had received formal education or training in PM&R during their medical studies. It emphasized how PM&R education was incorporated into medical education, illustrating its importance to the medical curriculum. With a mean value of 2.30 ± 0.464 and a significant p-value of 0.000, respondents also clearly distinguished between PM&R and physical therapy. It implied that responders had a nuanced grasp of the differences between these connected topics. The addressing of PM&R specialists was also viewed favorably, with a mean value of 3.93 ± 1.55 and a significant p-value of 0.000. It suggested that there was a cultural norm or shared understanding of proper professional conduct while engaging with PM&R specialists.

**Table 4:** Assessment of students' knowledge of the roles and responsibilities in Physical Medicine and Rehabilitation PM&R: Physical Medicine and Rehabilitation

Variables	Median	Mean ± SD	p-value
PM&R sub-specialities, etc.:	4.00	4.09 ± 1.429	0.000
Common conditions treated in PM&R:	4.00	3.89 ± 1.403	0.010
PM&R's role in patient care and recovery:	4.00	3.60 ± 1.373	0.003
Have you received formal education or training in PM&R during your medical studies?	2.00	2.15 ± 0.367	0.000
Do you differentiate between PM&R and Physical Therapy?	2.00	2.30 ± 0.464	0.000
How would you address a specialist in PM&R?	4.00	3.93 ± 1.55	0.000
In which clinical setting do you believe PM&R practices?	3.00	3.98 ± 1.98	0.286
Which demographics do you believe require the services of PM&R?	3.00	3.45 ± 1.14	0.000
Can patients of all ages consult a PM&R specialist?	4.00	3.53 ± 0.69	0.000
Do you think a referral from another doctor is necessary to see a PM&R physician?	3.00	3.15 ± 0.781	0.000
Is there a distinction between a Physiatrist, Podiatrist, and Psychiatrist?	4.00	4.34 ± 0.767	0.000
Do you believe PM&R only deals with patients who have already been diagnosed?	3.00	3.20 ± 0.807	0.000
Which conditions do you think PM&R physicians address?	2.00	3.21 ± 1.81	0.001
Do you understand the patient population that PM&R serves?	4.00	3.99 ± 1.066	0.000
Which bodily system is PM&R most concerned with?	6.00	4.86 ± 1.92	0.421

Further, a mean value of 3.45 ± 1.14 and a significant p-value of 0.000 in the study indicated that respondents believed certain populations needed PM&R services. It implied that there was a perceived target market for PM&R services. The results also showed a strong confidence (mean value 3.53 ± 0.69 and significant p-value 0.000) that patients of any age could see a PM&R specialist without a referral. It emphasized how easily accessible PM&R services were thought to be without requiring outside referrals (see Table 4).

Table 5 assessed students' attitudes about the duties and responsibilities in PM&R. The mean values with standard deviations (SD) and p-values gave insights into the students' viewpoints, while the median values gave an overview of the replies' central tendency. The findings showed that, with a median of 2.00, a mean of 2.01 ± 0.875, and a non-significant p-value of 0.849, most respondents had a neutral position about the inclusion of PM&R content in the undergraduate curriculum. It indicated that there was disagreement among students regarding the need for PM&R material in their curricula. The respondents' conviction that PM&R can improve patient's quality of life was largely in agreement, as indicated by the median score of 2.00, the mean score of 1.83 ± 0.377, and the non-significant p-value of 0.113. The tendency indicated a generally positive view regarding the prospective impact of PM&R on patient outcomes, even if it was not statistically significant. Responses to the issue of whether a PM&R physician should work in every multispecialty hospital showed a neutral posture, with a mean of 1.84 ± 0.364, a non-significant p-value of 0.359, and a median of 2.00. It suggested that there was not a broad agreement among students on the need for PM&R doctors in multispecialty hospital settings. The projected potential for PM&R as a specialization in the student's home country was found to be a noteworthy finding, with a mean of 2.41 0.678, a significant p-value of 0.000, and a median of 3.00. It suggested a more optimistic view of PM&R's continued existence as a legitimate and significant medical specialty. The respondents' level of confusion about the perceived knowledge of PM&R among medical professionals was 2.00 on the median, 1.93 ± 0.848 on the mean, and 0.038 on the marginally significant p-value scale. It implied that the medical community needed to become more conscious of and recognize PM&R. A significant correlation of 0.000 was found between undergraduate exposure and postgraduate plans incorporating PM&R, with a median of 3.00, a mean of 2.34 ± 0.736, and mean of 2.34. It highlighted the possible influence of early education on career choices by implying that exposure to PM&R during undergraduate studies may have influenced students' future career aspirations. There was variation among respondents on the possible role that the absence of PM&R departments and physicians at nearby hospitals played in its lower recognition; this was shown by a median score of 3.00, a mean score of 2.52 ± 0.658, and a non-significant p-value of 0.180. It showed that although the possible impact was acknowledged, perspectives regarding the actual impact of the infrastructure gap on recognition varied more (see Table 5).

**Table 5:** Assessment of students' attitude with the roles and responsibilities in Physical Medicine and Rehabilitation PM&R: Physical Medicine and Rehabilitation

Variables	Median	Mean ± SD	p-value
Should the undergraduate curriculum include content on PM&R?	2.00	2.01 ± 0.875	0.849
Do you believe PM&R can enhance patients' quality of life?	2.00	1.83 ± 0.377	0. 113
Do you agree that every multispecialty hospital should have a PM&R Physician?	2.00	1.84 ± 0.364	0.359
Is there potential for PM&R to be a specialty in your country?	3.00	2.41 ± 0.678	0.000
Do you consider PM&R a well-known speciality among the medical population?	2.00	1.93 ± 0.848	0.038
Is there a strong connection between undergraduate exposure and postgraduate plans involving PM&R?	3.00	2.34 ± 0.736	0.000
Could the lack of PM&R departments and physicians in your area's hospitals contribute to its lesser recognition?	3.00	2.52 ± 0.658	0. 18

## Discussion

The study discussed the knowledge and perception of medical students about PM&R at Qassim University. Concerning the issues regarding knowledge and perception of rehabilitation medicine, it was found that there is a moderate to low level of knowledge among medical students. Mostly, all concerned issues among medical students showed a significant relationship among them (P<0.05).

Medical students' lack of understanding is because they view PM&R differently than physiotherapy students do. According to our survey, 60.1% of medical students were knowledgeable about physical medicine and rehabilitation, but 69.8% of them were unable to distinguish between physical therapy and PM&R. It is comparable to the 2018 study by Tederko et al. that demonstrated medical students participate in preparatory instruction in specializations in which they may eventually practice medicine. Students studying physiotherapy responded at a rate of 61.2%, whereas students studying medicine responded at a rate of 75.9%. Although physiotherapy students seemed to be less aware of the rights of people with disabilities, the prevalence of impairment appeared to be evaluated by both groups with equivalent accuracy. Among students studying physiotherapy, non-response rates were greater. In Poland, there are currently no comprehensive solutions or norms governing the practice of rehabilitative medicine and physiotherapy by professionals. Certain medical specialties, like neurology and cardiology, assert that they can handle rehabilitation within their domains without the assistance of Physical and Rehabilitation Medicine (PRM) experts [15].

Our study results showed that 38.5% of medical students agreed to add PM&R courses at the undergraduate level. This study is comparable to one by Khosrawi et al. (2018), which found that students' inclination to choose physical medicine as a specialty was extremely low in 31.3% of cases and extremely high in 7.3% of cases. They concluded that adding a physical medicine course to the curriculum is essential to raising students' level of understanding in this field [1]. According to the results of the current study, the majority of medical students (84.4%) did not receive any official instruction or training while they were studying medicine. It is comparable to the research conducted in 2016 by Tederko et al., which looked at the variety of topics covered by the questionnaire and found that all respondent groups in central Europe had low levels of knowledge of PRM. What is especially concerning is how little PRMTs know about the fundamental ideas in their field of study. Inadequate PRM instruction in undergraduate and graduate medical education can be attributed to a number of factors, such as the absence of an internationally recognized curriculum and scope for PRM education [16], and unsatisfactory organization (education on disability and specific rehabilitation-related issues are only provided as part of training in rheumatology, family medicine, or geriatrics in graduate programs at some universities), insufficient funding, and a shortage of PRM-specialist university lecturers. Insufficient training results in a lack of expertise in overcoming disabilities, insufficient information about disabilities, an inability to diagnose disabilities, and an ignorance of PRM's potential [17].

Studies assessing medical students' understanding of PMR are scarce. Yorke's study suggested that in order to enhance patient care in this area, medical students' attitudes towards physical medicine need to be assessed and developed [12]. Our study results showed that 35.8% of medical students knew that the most concerning bodily system in PM&R is musculoskeletal. This study contradicts a study conducted on 217 general practitioners in Shiraz who were scheduled to attend a medical education course on low back pain. Following that, a questionnaire was used to assess these participants. Of those who answered, 56.8% stated that they had seen at least one patient with these issues in the previous month, and 92% felt that their knowledge of musculoskeletal issues was inadequate, particularly in the area of physical examinations. It runs counter to the findings of Raissi et al., who found that the majority of respondents thought musculoskeletal education was lacking in general practitioner training programs and that general practitioners identified musculoskeletal physical examination as the area that needed the greatest instruction [2]. The inadequate nature of fundamental rehabilitative training provided in medical schools was amply demonstrated by this study. The results indicate the areas of rehabilitation that general practitioners feel are most necessary and preferable, and it is important to take these into account when designing rehabilitation education programmes for Iranian medical students [1].

Our study results showed that 38.5% of medical students had agreed to the inclusion of PM&R courses for undergraduate students. A study by Faulk et al. had done a two-year study on 138 medical students. They used a two-week programme and administered pre-and post-tests to gauge students' attitudes and understanding of PMR. A little over 76% of them said that musculoskeletal education is crucial for medical students, and 42% said that the clinical curriculum needs to be designed with a longer time frame in mind [1,13]. According to a study by Khosrawi et al. (2018), the majority of students exhibited a high attitude and little awareness about PMR. The majority of students recommend physiatrists to patients with PMR. The findings of this study demonstrated a lack of understanding regarding PMR and its functions in the diagnosis and treatment of musculoskeletal system issues. As a result, the diagnosis and treatment of musculoskeletal system problems exhibited the lowest level of knowledge, while the electrodiagnostic test performance demonstrated the highest level of knowledge. It is similar to our study that found that 60.8% of medical students of Qassim University had agreed that a lack of PM&R is due to its less recognition in their hospital areas [1].

PMR is an essential part of medicine that has been aimed at achieving the goal of improving patients' independence in their lives. However, in Saudi Arabia, due to the lack of departments dealing with it in many areas and their hospitals, it is less popular as a medical school subject. Accordingly, our research estimated the medical students' level of knowledge and attitude about it. Intending to reinforce that side and provide good ways to gain many facts about this speciality. However, the study had some limitations. The study was conducted among 288 respondents in only one university, Qassim University. The sample size is too short to be generalized for the whole population. Another limitation is that the study included only medical students rather than comparing it with other medical fields to know about their knowledge and perception of PM&R.

It is recommended that the study be conducted among other medical universities and compared with other medical fields. It may be better to use the standard and international studies rather than locally made questionnaires so that they may be compared internationally. Furthermore, national and international standards for PRM training are required. It is also essential to support pertinent research. Postgraduate training in disability and rehabilitation should be required for physicians practising other specialities, especially family physicians. Increased curricular time devoted to musculoskeletal and physical examination skills might enhance physicians' knowledge, proficiency, confidence, and attitude toward musculoskeletal illnesses.

Strengths and limitations

This study offers valuable insights into the awareness and perceptions of PM&R among medical students at Qassim University. By leveraging a well-designed cross-sectional approach and utilizing validated questionnaire items, the study effectively captures students' knowledge and attitudes. The inclusion of students from both Unaizah Medical College and the main campus enhances the comprehensiveness of the findings within the context of Qassim University. Additionally, expert consultation in developing the questionnaire contributes to its content validity. However, the study’s focus on a single university may limit the generalizability of the results to other institutions or regions. The absence of a pilot study for the questionnaire might also impact the robustness of the findings. Addressing these aspects in future research could provide a more comprehensive understanding and strengthen the conclusions.

## Conclusions

The study concerning knowledge and perception of medical students regarding PM&R is prime to incorporate the PM&R curriculum at the undergraduate level. The study showed that there is a low to moderate level of knowledge about PM&R among medical students. Some students were familiar with PM&R but were unable to differentiate between PM&R and physical therapy. The study presents the need to train medical students in PM&R during their medical studies, develop more recognition, and integrate musculoskeletal and physical checkup skills among medical students. Moreover, it is recommended that it should have a practical implication across the nation so that more medical students develop knowledge and skills.
